# Regulation of the Rhizosphere Microenvironment by Arbuscular Mycorrhizal Fungi to Mitigate the Effects of Cadmium Contamination on Perennial Ryegrass (*Lolium perenne* L.)

**DOI:** 10.3390/microorganisms12112335

**Published:** 2024-11-15

**Authors:** Fan Yang, Jinji Han, Ruizhu Lin, Yuan Yin, Xiaoxia Deng, Yueming Li, Jixiang Lin, Jinghong Wang

**Affiliations:** College of Landscape Architecture, Northeast Forestry University, NO. 26 Hexing Road, Xiangfang District, Harbin 150040, China; yangfan1517@outlook.com (F.Y.); hanjinji0423@163.com (J.H.); 2023222302@nefu.edu.cn (R.L.); 2019224832@nefu.edu.cn (Y.Y.); dengxiaoxia@nefu.edu.cn (X.D.); 1258272709@nefu.edu.cn (Y.L.)

**Keywords:** Arbuscular mycorrhizal fungi (AMF), Cd contamination, *Rhizophagus irregularis* (*Ri*), *Lolium perenne* L., 16s rRNA sequencing technology

## Abstract

Rhizosphere microorganisms are crucial for enhancing plant stress resistance. Current studies have shown that Arbuscular mycorrhizal fungi (AMF) can facilitate vegetation recovery in heavy metal-contaminated soils through interactions with rhizosphere microbiota. However, the mechanisms by which AMF influences rhizosphere microbiota and plant growth under cadmium (Cd) stress remain unclear. In this study, *Lolium perenne* L. was inoculated with AMF (*Rhizophagus irregularis*) and grown in soils supplemented with Cd (0 mg kg^−1^, Cd0; 100 mg kg^−1^, Cd100). Plant biomass, antioxidant enzyme activities, peroxide content, Cd uptake, and rhizosphere bacterial community composition were evaluated. AMF inoculation reduced Cd influx in aboveground tissues, enhanced nutrient availability in the rhizosphere, and mitigated Cd biotoxicity. Additionally, AMF inoculation improved the scavenging efficiency of reactive oxygen species and alleviated oxidative stress in *L. perenne*, thereby mitigating biomass reduction. Moreover, AMF treatment increased leaf and root biomass by 342.94% and 41.31%, respectively. Furthermore, under the same Cd concentration, AMF inoculation increased bacterial diversity (as measured by the Shannon index) and reduced bacterial enrichment (as indicated by the ACE index). AMF promoted the enrichment of certain bacterial genera (e.g., *Proteobacteria* and *Actinobacteria*) in the Cd100 group. These findings suggest that AMF regulated the composition of the rhizosphere bacterial community and promoted the growth of potentially beneficial microorganisms, thereby enhancing the resistance of *L. perenne* to Cd stress. Cd contamination in soil severely limits plant growth and threatens ecosystem stability, highlighting the need to understand how AMF and rhizosphere microbes can enhance Cd tolerance in *L. perenne*. Therefore, inoculating plants with AMF is a promising strategy for enhancing their adaptability to Cd-contaminated soils.

## 1. Introduction

Heavy metal contamination, particularly from cadmium (Cd), poses significant global environmental hazards [1]. Among heavy metals and metalloids, Cd exhibits the highest percentage of soil samples (7.0%), exceeding the limit specified by the Ministry of Environmental Protection [2]. Notably, Cd exhibits strong mobility in the biosphere, posing significant risks to both soil and plants [3,4]. Cd, mainly existing in its divalent state, is released into the environment from various sources, including human activities, industrial emissions, volcanic eruptions, and other natural processes [5]. The high mobility of Cd in soil facilitates easy absorption by plant roots, leading to rapid translocation throughout the plant and significant accumulation in the aboveground parts of vegetation [6]. This accumulation can reduce plant biomass and lead to Cd buildup in plants [7]. Therefore, addressing Cd pollution is crucial.

Arbuscular mycorrhizal fungi (AMF) can enhance plant adaptability to challenging environmental conditions through both direct and indirect mechanisms. AMF directly forms an extensive mycelial network around the roots of the host plant, creating a reciprocal exchange system in which plant-derived carbon is traded for essential nutrients such as nitrogen and phosphorus. This symbiosis enables AMF to utilize 4% to 20% of the total photosynthetic output of plants to facilitate their metabolic processes, thereby promoting plant growth and development in a positive feedback loop [8]. To indirectly mitigate heavy metal toxicity, AMF release specific effector proteins and peptides that reduce the mobility and bioavailability of heavy metals in the soil. This process modulates the uptake and distribution of heavy metal ions in both plants and the surrounding environment [9,10]. AMF sequester significant amounts of Cd within their spores and vesicles, thereby effectively reducing environmental heavy metal concentrations, limiting Cd translocation from roots to aboveground tissues, and alleviating heavy metal stress on plants [11]. Additionally, AMF enhance rhizosphere community structure and diversity, strengthen interspecies interactions, and attract beneficial microbiota to optimize nutrient acquisition from the soil, further promoting plant growth [12].

*Lolium perenne* L. is a vital pasture and forage species commonly found in temperate regions and native to Europe and was introduced into China as a lawn grass in the 1930s [13]. The *L. perenne* used in this study is a high-quality cool-season turfgrass variety that is widely cultivated in urban areas of temperate and cool climate regions in China, including the northeast, southwest, and eastern regions [14]. *L. perenne*, a perennial monocot in the Poaceae family, is highly valued for its ease of cultivation, high biomass yield, excellent regenerative capacity, and strong resistance to pests and diseases [15]. The economic and ecological significance of *L. perenne* is well recognized, particularly for its role as a vital turfgrass and green manure crop [16]. Notably, ryegrass exhibits a high tolerance to heavy metals and can accumulate these contaminants, making it an ideal candidate for phytoremediation, particularly in turf management on golf courses and in heavy-metal polluted areas [17,18].

This study investigated the effectiveness of AMF, specifically *Rhizophagus irregularis*, in mitigating plant growth inhibition in heavy metal-contaminated soils [19]. Previous studies have shown that *Rhizophagus irregularis* reduces the toxicity of Cd in the soil, thereby altering the rhizosphere soil microbial community, alleviating Cd-induced damage to rice, and enhancing plant tolerance to heavy metal stress. In this study, pot experiments were conducted to artificially inoculate *Rhizophagus irregularis* to evaluate its potential impact on the rhizosphere microbial community and its ability to reduce Cd toxicity in *L. perenne*. Plant growth and ectopic Cd accumulation were measured to assess the responses of the rhizosphere microbial community.

## 2. Materials and Methods

### 2.1. Experimental Design

The experimental soil was collected from farmland in Qiqihar City at a depth of 20–40 cm. The soil had an organic matter content of 33.9 g/kg, total nitrogen of 2.97 g/kg, hydrolyzed nitrogen of 73.5 g/kg, available phosphorus (AP) content of 5.06–9.38 g/kg, and a pH of 6.2. The soil was sieved through a 2 mm mesh and then mixed with river sand in a 1:1 (v) ratio. Cd treatment concentrations in the culture matrix were set at 0 mg/kg (Cd0) and 100 mg/kg (Cd100) using CdCl_2_·2.5H_2_O. To achieve a final Cd concentration of 100 mg/kg, 223.46 mg of CdCl_2_·2.5H_2_O was dissolved in 1.20 L of autoclaved 1/4 Hoagland solution (autoclaved at 121 °C for 2 h). The cadmium-free group (control) was treated with an equivalent volume of Hoagland solution without the addition of CdCl_2_·2.5H_2_O. Water was circulated three times to stabilize the matrix. The culture matrix was then transferred into plastic pots (12.5 cm in diameter and 11.5 cm in height), each containing 1000 g of the matrix. Subsequently, inactivated and activated AMF (*Rhizophagus irregularis*) inoculum (provided by the College of Landscape Architecture, Yangtze University) was added at a rate of 50 g per pot. Fifty *L. perenne* seeds were planted in each pot. After germination, the seedlings were thinned to 10 plants per pot.

Plants were grown for 55 days under greenhouse conditions at 28/22 °C (day/night), with a photon flux density of 350 µmol m^−2^ s^−1^ (natural sunlight combined with cool white fluorescent light for 16 h per day) and relative humidity of 80–85%. To maintain soil moisture at 80% of the soil water-holding capacity, plants were regularly irrigated with deionized water. A 1/4 strength Hoagland solution was applied weekly to ensure adequate nutrient supply. After 55 days of growth, the whole leaves, roots, and rhizosphere soil of *L. perenne* samples were collected from all four treatments. Fresh biomass and plant height were measured, and net increases in growth and height were determined. For each treatment, five sample replicates were taken, with a subsample of fresh material from each plant part collected, frozen in liquid nitrogen, and stored at −80 °C for later analysis. The remaining samples were dried at 60 °C for 72 h until they reached a constant weight. Once dried, the samples were ground into powder using a mini-vegetation disintegrator (FZ102; Tianjin City Test Instrument Co., Ltd., Tianjin, China). Vermiculite that remained attached to the root segments after gentle shaking was classified as rhizosphere sediment, air-dried, and passed through a 10-mesh sieve [20].

Thus, the total four treatments included *L. perenne* inoculated with AMF (AMCd0) or without AMF (NMCd0) under Cd-free conditions and *L. perenne* inoculated with AMF (AMCd100) or without AMF (NMCd100) under 100 mg/kg Cd^2^⁺ supplementation. Each pot contained 10 *L. perenne* plantlets, with five replicates per treatment (4 treatments × 5 replicates = 20 pots). The experimental design included five replicates to assess metal accumulation and physiological growth responses in *L. perenne*, while three randomly selected replicates were used to examine rhizosphere bacterial communities. Root fragments (~1 cm in length) were randomly collected from the harvested root material of each plant across all four treatments. These fragments were first immersed in a 10% (w/v) KOH solution for 30 min in a water bath at 90 °C, followed by staining with 0.5% acid fuchsin. The extent of AMF colonization was evaluated using the gridline intersect method, with 200–300 intersects (*n* = 5) [21].

### 2.2. Testing of the Antioxidant System

Fresh 0.1 g samples of *L. perenne* leaf or root tissues were ground into a fine powder using liquid nitrogen, then homogenized at 4 °C with 1 mL of extraction buffer from reagent kits. The levels of malondialdehyde (MDA), oxygen-free radicals (OFR), and the activities of Catalase (CAT) and Peroxidase (POD) were measured using commercial assay kits (Suzhou Comin Biotechnology Co., Ltd., Suzhou, China) [22].

### 2.3. Physiological Parameter Measurement

Leaf, stem, and root samples (0.5 g) were dried, weighed, and then subjected to wet digestion with a mixture of HNO_3_ and HClO_4_ (4:1, *v*/*v*) [23]. Cadmium (Cd) concentration was measured using atomic absorption spectrophotometry (AAS; ZA-3000, Hitachi, Chiyoda-ku, Tokyo, Japan). Quality control was performed using seaweed (GBW 10023, Institute of Geophysical and Geochemical Exploration, Langfang, China) as the standard reference material, with accuracy within 100 ± 20% [24].

Five grams of rhizosphere sediments were mixed with 40 mL of diethylenetriaminepentaacetic acid (DTPA) extraction solution (0.1 M triethanolamine, 10 mM CaCl_2_, 5 mM DTPA, pH 7.3), shaken at 180 rpm for 2 h, and then centrifuged at 5000× *g* for 10 min. The supernatant was collected and filtered and the DTPA-extractable cadmium (Cd) concentration was determined as previously described (*n* = 5). Metal concentrations in plant tissues and soils were used to calculate the translocation factor (TF) and bioconcentration factor (BCF) [25]. The formula is as follows:

BCF=(Cd in plant/Cd in substrate)

TF=(Cd in shoots/Cd in roots)

According to the China National Standards (GB 5009.91–2017, GB 5009.241–2017) [26,27], 1 g of powdered tissue samples (leaves and roots) from each treatment was weighed and wet digested with a HNO_3_:HClO_4_ (9:1, *v*/*v*) mixture [28]. Nutritional elements, including potassium (K) and magnesium (Mg), were analyzed by atomic absorption spectrophotometry (*n* = 5) [29]. Nitrogen (N) and phosphorus (P) concentrations in dried soil were also measured. Ammonium nitrogen (AN) and nitrate nitrogen (NN) were extracted from plant tissues using the Bremner micro-Kjeldahl method, while phosphorus was extracted by wet oxidation with nitric acid and perchloric acid [30]. Total phosphorus was determined spectrophotometrically at 420 nm using the molybdate and acid per-sulfate digestion method.

### 2.4. 16S rRNA Sequencing

In this experiment, microbial DNA from rhizosphere soil was extracted using the FastDNA SPIN Kit for Soil (Qbiogene Inc., Carlsbad, CA, USA) following the manufacturer’s protocol [31]. Soil microbial sequencing was performed by Majorbio Biopharm Technology (Shanghai, China). DNA quality and concentration were assessed by 1.0% agarose gel electrophoresis and using a NanoDrop ND-2000 spectrophotometer (Thermo Fisher Scientific, Waltham, MA, USA). The extracted DNA was stored at −80 °C for future use.

The targeted microbial regions for sequencing were 515F (GTGYCAGCMGCCGC-GGTAA) and 907R (CCGTCAATTCMTTTRAGT), amplified using the ABI GeneAmp 9700 PCR thermal cycler (ABI, Foster City, CA, USA) [32]. After preliminary tests, PCR amplification was carried out with TransGen AP221-02: TransStart FastPfu DNA Polymerase in a 20 μL reaction volume [33]. The PCR conditions were initial denaturation at 95 °C for 3 min, followed by 27 cycles of denaturation at 95 °C for 30 s, annealing at 55 °C for 30 s, extension at 72 °C for 45 s, and a final extension at 72 °C for 10 min, with the process ending at 4 °C. Each soil sample was processed in triplicate.

PCR products were extracted from a 2% agarose gel, pooled by sample, and re-analyzed by gel electrophoresis. The products were purified using the AxyPrep™ DNA Gel Extraction Kit (Axygen, Union City, CA, USA) [34]. Quantification was performed using the QuantiFluor ST Blue fluorescence quantification system (Promega, Madison, WI, USA) based on initial electrophoresis results. Paired-end (PE) reads were sequenced by Illumina (San Diego, CA, USA), spliced, and subjected to quality control and filtering [34].

### 2.5. Statistical Analysis

The significant differences between groups were evaluated using one-way analysis of variance [35]. Principal coordinate analysis (PCoA) was conducted based on the Bray-Curtis dissimilarity to assess relationships among groups using operational taxonomic units (OTUs). The resulting spatial relationships were visualized using the “vegan” package (v2.4.3) in R [36]. Differential analyses of microbial phyla and species were performed using the “kruskal. test” function from the “stats 4.48” package [37]. Co-occurrence network analysis for microbial data was performed with the “igraph 2.1.1” package in R to construct networks using Spearman correlations [38], and networks were visualized in Gephi (version 0.10) [39]. Data visualization was performed with the “ggplot2 3.5.1” package to produce high-quality graphics [40]. All statistical analyses were conducted in R version 3.6.2.

## 3. Results

### 3.1. AMF Colonization and Its Impact on Biomass, Nutrients, and Cadmium in Soil and Plant

**Figure 1:**
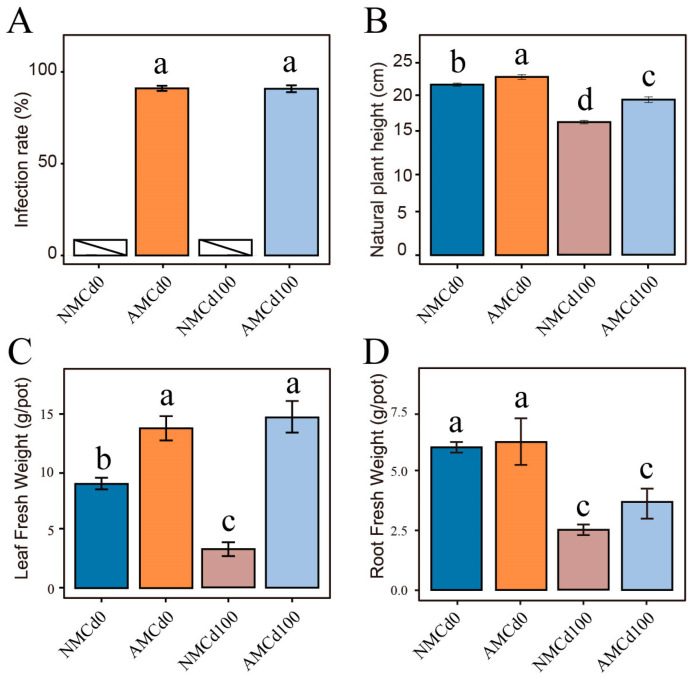
Growth and infection of *L. perenne* under different soil treatments. (**A**) Infection rate, (**B**) Plant height, (**C**) Shoot fresh weight, and (**D**) Root fresh weight of *L. perenne* per pot. Data are presented as Mean ± SEM, *n* = 5. AM and NM represent with and without AMF inoculation, respectively. Cd0 indicates no cadmium, while Cd100 represents 100 mg/kg Cd. Statistical significance was determined by one-way ANOVA, with significance indicated by different letters.

The uninoculated treatment groups did not exhibit AMF colonization. However, the inoculated groups exhibited colonization rates ranging from 83.23% to 92.83%. Notably, no significant difference was observed between the colonization rates of the AMCd0 (85.7%) and AMCd100 groups (90.12%) (*p* > 0.05) (Figure 1A). In the NMCd0 and NMCd100 groups, exposure to Cd100 significantly reduced the shoot fresh weight, root fresh weight, and plant height of *L. perenne* by 342.94%, 41.31%, and 23.21%, respectively, compared with those of Cd0 (*p* < 0.05) (Figure 1B–D). In the Cd100 treatment group, inoculation with AMCd100 significantly increased plant height and root fresh weight by 4.12% and 15.09%, respectively, compared with those of NMCd100. Moreover, AMCd100 significantly affected the aboveground fresh weight of the plant by 508.79% (*p* < 0.05) (Figure 1B–D).

**Figure 2:**
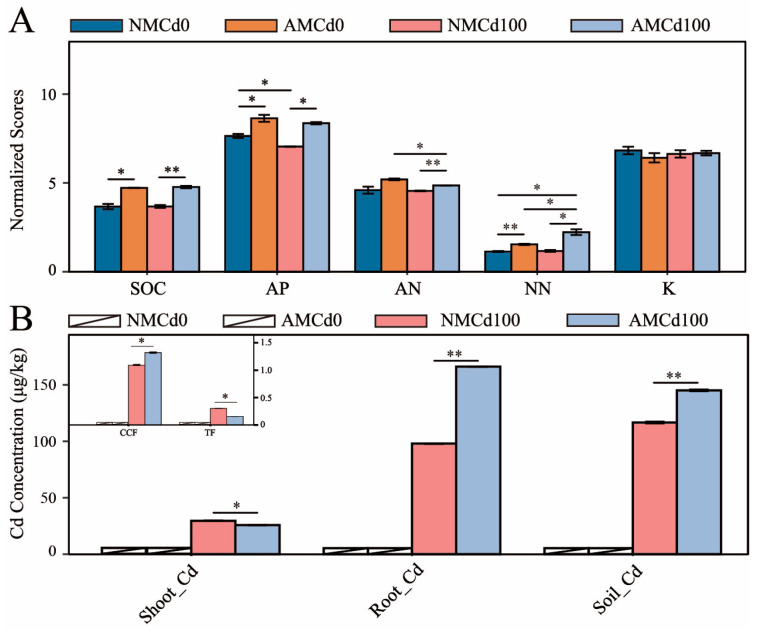
Nutrient content in rhizosphere soil and cadmium (Cd) levels in plants under different treatments. (**A**) C content, ammonia nitrogen (AN), nitrate nitrogen (NN), potassium (K), and magnesium (Mg) in rhizosphere soil. (**B**) Accumulation of Cd in different plant tissues under various treatments, along with bioconcentration factors (BCFs) and translocation factors (TFs). AM and NM represent with and without AMF inoculation, respectively. Cd0 indicates no Cd, while Cd100 represents 100 mg/kg Cd. Data are presented as mean ± SEM, *n* = 5 biological replicates. Statistical significance was determined by one-way ANOVA: * *p* < 0.05, ** *p* < 0.01.

Different treatments influenced nutrient content in the rhizosphere soil of *L. perenne* (Figure 2A). The Cd treatment significantly reduced AP by 7.82% in the NCd100 group compared to the NCd0 group (*p* < 0.05). In the NMCd100 and AMCd100 groups, AMF inoculation significantly increased soil carbon (C) by 2.48%, AP by 4.47%, ammonium nitrogen (AN) by 8.78%, and nitrate nitrogen (NN) by 47.68% (*p* < 0.05). This suggests that AMF inoculation improved nutrient availability even under Cd contamination.

Inoculation with AMCd100 increased the Cd content in the roots and rhizosphere soil by 69.69% and 24.45%, respectively, but reduced leaf Cd content by 12.89% (*p* < 0.05) (Figure 2B). To evaluate Cd transport and enrichment, the bioconcentration factor (BCF) and translocation factor (TF) were calculated (Figure 2B). Compared with NMCd100, AMF inoculation (AMCd100) significantly increased BCF by 20.98% and reduced TF by 48.67% (*p* < 0.05).

### 3.2. Reactive Oxygen Species (ROS) and Peroxidase Activities

**Figure 3:**
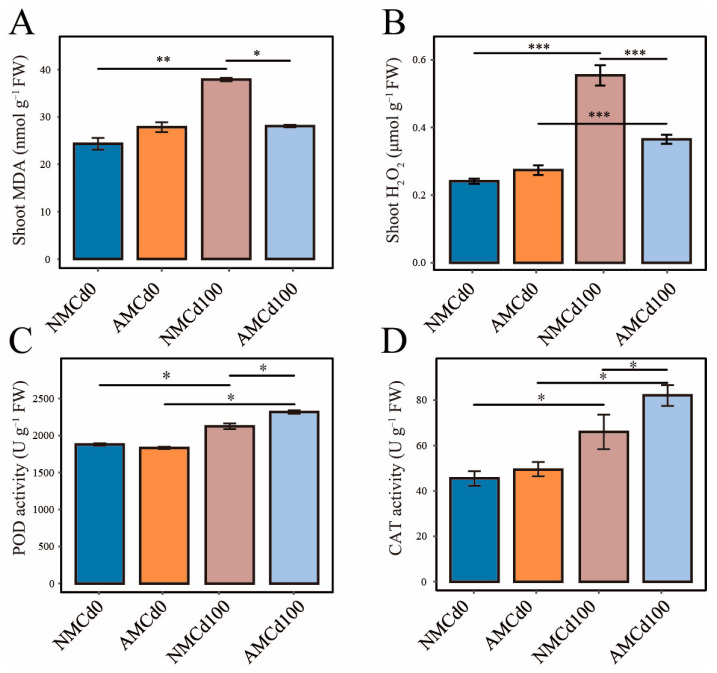
Response of the antioxidant system in *L. perenne* to different treatments. (**A**) Malondialdehyde (MDA) content, (**B**) Hydrogen peroxide (H_2_O_2_) content, (**C**) Peroxidase (POD) activity, (**D**) Catalase (CAT) activity. Data are presented as mean ± SEM, *n* = 5 biological replicates. AM and NM represent with and without AMF inoculation, respectively. Cd0 indicates no cadmium, while Cd100 represents 100 mg/kg Cd. Statistical significance was determined by one-way ANOVA: * *p* < 0.05, ** *p* < 0.01, *** *p* < 0.001.

Changes in plant antioxidant compounds and antioxidant enzyme activities were examined under different Cd treatments, including malondialdehyde (MDA) content, H_2_O_2_ content, Peroxidase (POD) activity, and Catalase (CAT) activity (Figure 3). Cd treatment (NMCd100) caused a significant increase in MDA and H_2_O_2_ contents by 55.57% and 113.12%, respectively, in the NMCd100 group compared with the NMCd0 group (*p* < 0.05) (Figure 3). However, in the AMCd100 group, AMF inoculation significantly reduced the MDA and H_2_O_2_ contents by 25.90% and 29.81%, respectively (*p* < 0.05) (Figure 3). The activities of POD and CAT enzymes followed the same trend as the peroxidation products.

### 3.3. Microbial Composition in Rhizosphere Soil

**Figure 4:**
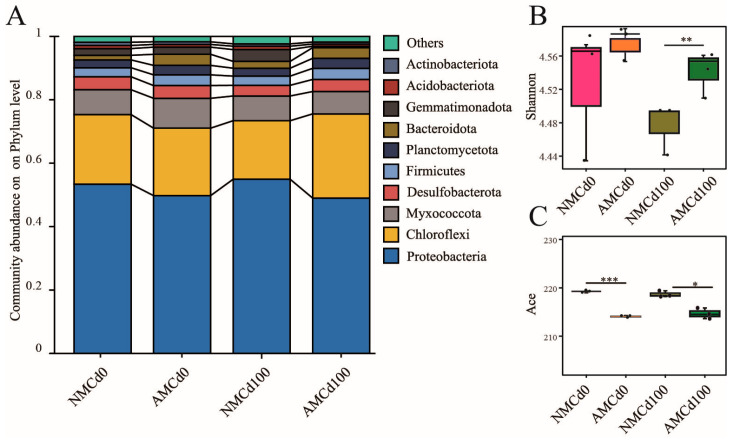
Effects of Cadmium (Cd) concentration and Arbuscular mycorrhizal fungi (AMF) inoculation on rhizosphere soil bacterial communities. (**A**) Community abundance at the phylum level across different treatments. (**B**) the Shannon index. (**C**) the ACE index. AM and NM represent with and without AMF inoculation, respectively. Cd0 indicates no Cd, while Cd100 represents 100 mg/kg Cd. Statistical significance was determined by one-way ANOVA: * *p* < 0.05, ** *p* < 0.01, *** *p* < 0.001.

The diversity analysis of the 12 samples revealed a total of 36 phyla, 106 classes, 237 orders, 388 families, 680 genera, 1313 species, and 3812 OTUs for bacterial taxonomic annotation (Table A1). We examined community abundance at the phylum level across different treatments, including uninoculated (NMCd0 and NMCd100) and AMF-inoculated (AMCd0 and AMCd100) groups (Figure 4A). *Proteobacteria*, comprising 42.66–51.84% in all treatment groups, was the dominant phylum, followed by *Chloroflexi* and *Myxococcota*. Alpha diversity analysis revealed that AMF inoculation under Cd contamination altered the richness and diversity of the rhizosphere microbial community in *L. perenne* (Figure 4B,C). The Shannon index significantly increased by 22.51% in the AMCd100 group compared with NMCd100 (*p* < 0.01). Conversely, the ACE index decreased significantly by 3.60% and 3.66% in the AMCd0 and AMCd100 groups, respectively, compared with NMCd100 (*p* < 0.01). 

**Figure 5:**
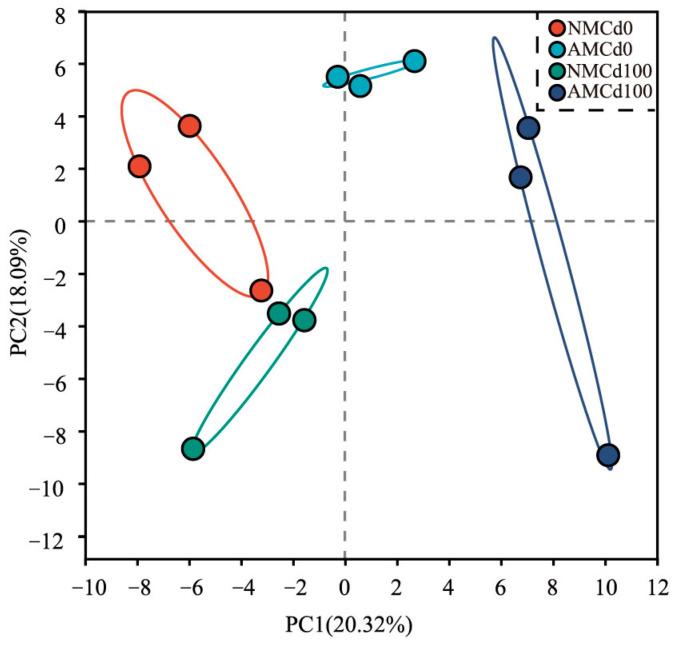
PCoA of rhizosphere soil bacterial communities under different Cd concentrations and AMF inoculation treatments. AM and NM represent with and without AMF inoculation, respectively. Cd0 indicates no Cd, while Cd100 represents 100 mg/kg Cd.

Figure 5 shows the PCoA results of the rhizosphere soil bacterial community under different Cd treatments and AMF inoculation. The first two principal coordinates (PC1 and PC2) accounted for 20.32% and 18.90% of the total variation, respectively. Under AMF inoculation, the AMCd0 and AMCd100 groups were separated from the NMCd0 and NMCd100 groups along the *Y*-axis. Under Cd treatment, the NMCd0 and AMCd0 groups were separated from the NMCd100 and AMCd100 groups along the *X*-axis (Figure 5).

### 3.4. Rhizosphere Nutrient Efficiency and Plant Cadmium Uptake

**Figure 6:**
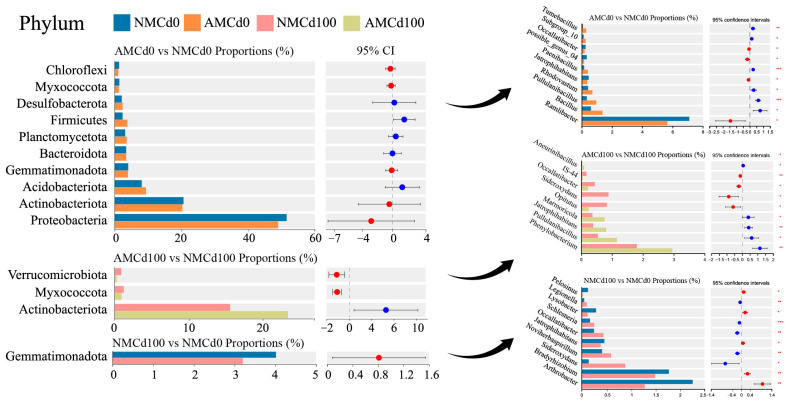
Comparison of relative abundance of phyla and genera of rhizospheric bacteria in *L. perenne* among. treatments. AM and NM represent with and without AMF inoculation, respectively. Cd0 indicates no cadmium, while Cd100 represents 100 mg/kg Cd. Bar graphs display the proportional abundance of major taxa, while adjacent forest plots illustrate the 95% confidence intervals (CIs) for the effect sizes of differential abundance. Statistical significance was determined by one-way ANOVA: * *p* < 0.05, ** *p* < 0.01, *** *p* < 0.001.

Species difference analysis revealed significant variations in microbial species at both the phylum and genus levels in the rhizosphere of *L. perenne* (Figure 6). At the phylum level, the relative abundance of dominant bacterial phyla significantly varied across treatments. The relative abundances of *Proteobacteria* and *Actinobacteria* decreased by 2.733% and 2.048%, respectively, in the AMCd0 group compared with the NMCd0 group. In contrast, the relative abundance of *Actinobacteriota* significantly increased by 50.20% in the AMCd100 group compared with the NMCd100 group owing to AMF inoculation. In treatment groups without AMF inoculation, the relative abundance of *Gemmatimonadota* was 3.202% in the NMCd100 group and 4.017% in the NMCd0 group (Figure 6).

At the genus level, in groups without Cd treatment, the AMCd0 group exhibited significantly lower relative abundances of *Ramlibacter*, *Jatrophihabitans*, *possible_genus_04*, and *Occallatibacter* than the NMCd0 group (*p* < 0.05) (Figure 6). Notably, AMF inoculation reduced the relative abundance of *Ramlibacter* by 20.34%. In contrast, the AMCd0 group exhibited significantly higher relative abundances of *Bacillus*, *Pullulanibacillus*, *Rhodovastum*, *Paenibacillus*, and *Tumebacillus*. Particularly, the AMCd0 group contained *Tumebacillus*, a unique genus, accounting for 0.2943% of the total microbial community (*p* < 0.05) (Figure 6). Under Cd treatment, the AMCd100 group exhibited a relative abundance of *Phenylobacterium* at 3.1%, representing a 2.3% increase compared with the NMCd100 group (*p* < 0.05) (Figure 6). Similarly, the abundance of *Jatrophihabitans* increased by 1.1% in the AMCd100 group, reaching 1.5%, while the abundance of *Aneurinibacillus* decreased by 1.2%, from 1.9% in the NMCd100 group to 0.7% (*p* < 0.05) (Figure 6). Additionally, the relative abundance of *Arthrobacter* in the NMCd100 group increased by 1.5%, reaching 2.1%, compared with the NMCd0 group (*p* < 0.05) (Figure 6). Furthermore, the abundance of *Bradyrhizobium* increased by 1.2% in the NMCd100 group, reaching 1.9%, while the abundance of *Sideroxydans* increased by 0.8%, from 0.5% in the NMCd0 group to 1.3% (*p* < 0.05) (Figure 6).

**Figure 7:**
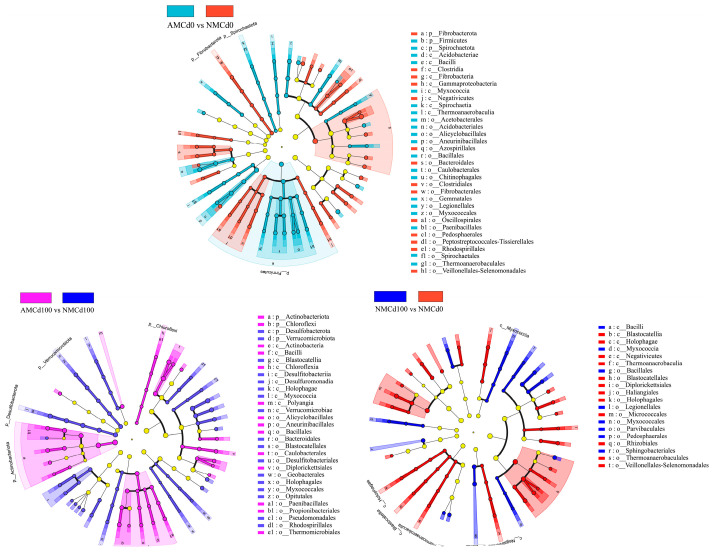
LEfSe analysis (LDA > 2) of bacterial taxa under different treatment conditions in the rhizosphere soil. AM and NM represent with and without AMF inoculation, respectively. Cd0 indicates no cadmium, while Cd100 represents 100 mg/kg Cd.

The LEfSe analysis revealed significant changes in the bacterial community composition in the rhizosphere under different treatments (Figure 7). Compared with AMCd0 and NMCd0, the AMCd0 group had significantly higher abundances of the bacterial phyla *Fibrobacterota* and *Firmicutes*. In contrast, the NMCd0 group exhibited a higher abundance of *Acidobacteriota* and *Spirochaetota*, indicating that bacterial communities responded differently to AMF inoculation in the absence of Cd. A comparison between AMCd100 and NMCd100 treatments revealed that AMCd100 had a significantly higher abundance of *Actinobacteriota* and *Verrucomicrobiota*, while NMCd100 featured a higher abundance of *Myxococcota* and *Gemmatimonadota*. This suggests that AMF inoculation promoted the growth of beneficial microbes associated with stress tolerance, thereby altering bacterial composition under high Cd stress. Moreover, the NMCd100 group exhibited higher enrichment of *Actinobacteriota* and *Thermomonosporaceae*, while NMCd0 had a higher abundance of *Bacilli* and *Clostridia*. These findings indicate that Cd exposure significantly altered the microbial community in the absence of AMF inoculation, potentially reducing microbial diversity and favoring stress-tolerant taxa.

**Figure 8:**
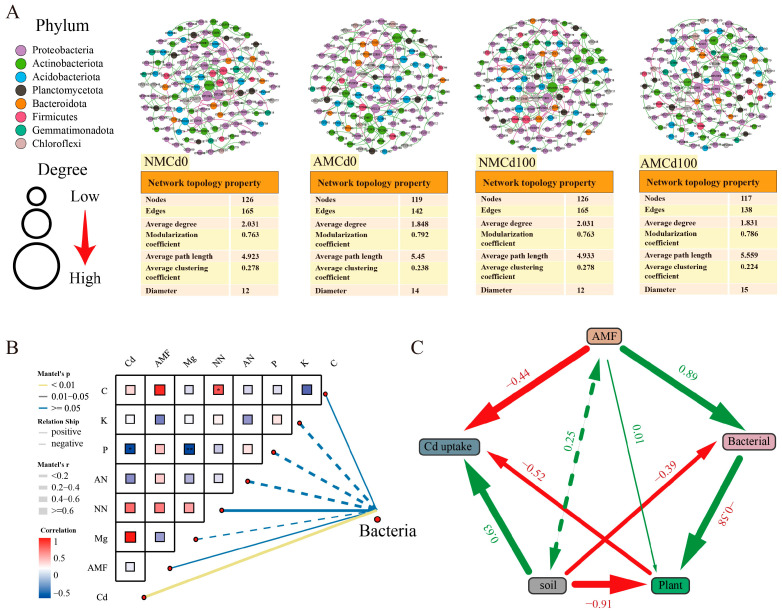
Microbial community structure and network interactions under cadmium (Cd) stress. AM and NM represent with and without AMF inoculation, respectively. Cd0 indicates no Cd, while Cd100 represents 100 mg/kg Cd. (**A**) Co−occurrence network analysis of microbial communities across treatment groups, with nodes representing taxa, color-coded by phylum, and node size indicating connectivity. Key metrics (modularity and clustering coefficient) are summarized below. (**B**) Correlation matrix showing relationships between environmental variables and bacterial community structure. Significant correlations (Mantel test, *p* < 0.05) are marked, with the color gradient reflecting strength and direction. (**C**) Structural equation model (SEM) illustrating interactions among Cd (content, translocation coefficient, and accumulation coefficient), soil nutrient content, plant parameters (biomass and antioxidant index), bacterial metrics (OTU abundance and diversity), and AMF (inoculation rate). Arrows indicate influence direction, with line thickness proportional to path coefficients; positive relationships are in green, and negative relationships are in red. Statistical significance was determined by one-way ANOVA: * *p* < 0.05; ** *p* < 0.01.

Compared with the uninoculated group, the AMF-inoculated group had fewer nodes and edges in the bacterial network, with 119 and 117 nodes in AMCd0 and AMCd100, respectively (Figure 8A). However, at high Cd concentration (Cd100), the AMF-inoculated group (AMCd100) exhibited a higher modularity coefficient and edge diameter (0.786 and 15) than the uninoculated group (NMCd100) (0.763 and 12). *Proteobacteria* and *Actinobacteria* were significantly enriched in all four treatment groups. Particularly, AMCd100 exhibited high abundances of *Allorhizobium*, *Neorhizobium*, *Pararhizobium*, *-Rhizobium*, *Massilia*, and *Phenylobacterium* at 6.14, 5.28, and 4.81, respectively.

The Mantel test and Spearman correlations were used to examine the relationships between soil factors and bacterial groups involved in Cd passivation, particularly focusing on Cd, soil properties, and microorganisms (Figure 8B). Mantel correlation analysis was performed to explore the potential relationships between various environmental factors and rhizosphere bacterial communities. The results revealed a significant negative correlation between Cd and the bacterial community (Mantel’s r > 0.6, *p* < 0.01), suggesting that Cd contamination strongly disrupted the bacterial community structure. In contrast, carbon (C) and phosphorus (P) exhibited positive and statistically significant correlations with the bacterial community (*p* < 0.05), indicating that these soil nutrients may enhance bacterial community composition and diversity. Although AMF featured a positive correlation with the bacterial community, this relationship was not statistically significant (*p* > 0.05). Other environmental factors, including potassium (K), magnesium (Mg), NN, and AN displayed weaker and non-significant correlations with bacterial communities.

We used structural equation modeling (SEM) to analyze the effects of AMF on plant growth, soil nutrition, and Cd uptake and translocation (Figure 8C). The SEM results revealed that AMF inoculation, directly and indirectly, influenced both microbial community structure and diversity, which affected microbial functions, plant growth, and Cd absorption and transport through changes in soil nutrients. Among these, the correlation coefficient between AMF and bacteria was 0.89, and the correlation coefficient between AMF and Cd absorption was −0.44.

## 4. Discussion

Cd is a highly toxic heavy metal with high mobility in soil, which poses a significant risk of soil pollution, particularly in northeast China [41]. Cd accumulation can significantly reduce soil nutrient content, thereby inhibiting plant growth. This study indicates that the contents of key soil nutrients, including NN, AN, P, and K, significantly decreased under Cd contamination. This reduction may be attributed to Cd binding with these nutrients, forming stable chemical compounds that hinder plant absorption [42]. Additionally, Cd can disrupt the protein structure of enzymes, further reducing the availability of vital nutrients in the soil and leading to a decline in soil quality [43]. Conversely, under Cd contamination, the soil organic carbon content significantly increased owing to the secretion of organic metabolites by plant roots, which promotes the growth and reproduction of soil microorganisms [44]. These microorganisms enhanced the nutritional status of the microenvironment, promoting the formation of complex microbial communities [44]. Yuan, et al. [45] found that Cd-contaminated soils harbored Cd-resistant microbial communities. These microorganisms utilized mechanisms such as biosorption and bioconcentration to effectively remove large amounts of Cd from the soil. Moreover, the microorganisms can transform Cd into non-toxic or less toxic forms [46]. Furthermore, the microorganisms released organic matter during their metabolic processes, contributing to soil organic matter accumulation and enhancing the soil microenvironment [47]. The toxic effects of heavy metals can disrupt the metabolic activities and growth regulation of plants, which affects their growth rate and seedling development [48]. Research has shown that high Cd concentrations in soil can hinder plant growth and reduce physiological functions [49].

This study found that AMF inoculation significantly reduced Cd content in the aerial parts of *L. perennial* but increased Cd accumulation in the roots and rhizosphere soil, thereby promoting plant growth. Cd exposure significantly increased MDA and H_2_O_2_ levels in plants, leading to oxidative stress. The Cd-treated control group (NMCd100) exhibited significantly higher MDA levels. However, the AMCd100 group had lower MDA levels, indicating that AMF inoculation mitigated Cd-induced lipid peroxidation. Similarly, the Cd-treated control group exhibited significantly higher H_2_O_2_ levels, but AMF-treated groups had significantly lower H_2_O_2_ levels [50]. The extensive network of intra- and extraradical hyphae formed by AMF directly sequestered Cd, which significantly limited its upward translocation in plants [51]. Additionally, AMF produced glomalin-related soil protein, a glycoprotein that binds and stabilizes metals, which facilitated Cd immobilization within the rhizosphere soil and root tissues, thereby reducing its transfer to the shoots [52]. The significant increase in BCF and decrease in TF further supported this mechanism. AMF inoculation reduced Cd concentration in the aerial parts, increased biomass, and alleviated oxidative stress, thereby effectively mitigating Cd toxicity in plants.

Under Cd contamination, the rhizosphere soil microbial community at the phylum level exhibited significant changes after inoculation with AMF (Figure 4). Notably, *Actinobacteriota* emerged as the dominant phylum across all treatment groups, particularly in the AMF-inoculated groups (AMCd0 asnd AMCd100), with a significant increase in their relative abundance [8]. A previous study has revealed that a combination of *actinomycetes* and mucor can clean up contaminated soil, particularly zinc, lead, and manganese compounds [52].

At the genus level, *Phenylobacterium*, *Pullulanibacillus*, *Jatrophihabitans*, and *Marmoricola* were significantly enriched in the AMCd100 group. These genera may promote growth mechanisms in plants, such as nutrient absorption and water balance, which can enhance plant tolerance and promote root system development, potentially mitigating the effects of Cd contamination [53]. However, microbial interaction can be complex, with the effects on plants varying based on environmental factors and competitive or symbiotic relationships [54]. Therefore, in applying beneficial microorganisms such as AMF to improve plant adaptability to Cd contamination, it is crucial to consider various factors such as soil conditions and plant varieties to achieve optimal outcomes [55]. For example, studies have shown that different soil conditions can influence the effectiveness of AMF-plant interactions in Cd-contaminated environments. In acidic soils, AMF can significantly enhance the Cd tolerance of plants and reduce Cd accumulation in plant tissues, but this effect may be weaker in alkaline soils [56]. Moreover, plant species exhibit varying levels of Cd tolerance after AMF inoculation. For example, maize varieties differ in their Cd uptake and utilization after treatment with AMF [57]. This highlights the importance of selecting suitable plant species to enhance the effectiveness of AMF applications.

Symbiotic network analysis revealed that AMF inoculation (AMCd0 and NMCd100) reshaped the rhizosphere microbial community. The results indicated that AMF inoculation promoted an increase in plant-associated growth-promoting bacteria, which provided vital organic compounds to AMF. This interaction created a more favorable environment for both the fungi and the plant. Consequently, the synergistic relationship between AMF and rhizosphere microorganisms stimulated root growth in *L. perenne*, enhanced nutrient uptake from the soil, and contributed to an overall improvement in plant growth rate and quality [54,58].

Mental tests and SEM analysis revealed that plant physiological and soil nutritional indicators significantly influenced the abundance and distribution of microbial species. SEM results indicated the complex relationships between AMF infestation, soil nutrients, bacterial communities, plant growth, and Cd uptake and transport. AMF inoculation significantly promoted bacterial diversity and improved soil quality, thereby enhancing Cd uptake by plants. However, AMF directly reduced Cd translocation through mechanisms such as chelation or sequestration. Improved soil quality positively affected plant growth, while bacterial communities had a minimal negative impact on plant growth owing to resource competition [59]. Overall, AMF played a critical role in regulating both microbial interactions and Cd dynamics, which aided in mitigating Cd contamination and improving nutrient uptake.

The synergistic effects of AMF and rhizosphere microorganisms can significantly enhance nutrient uptake, promote growth under adverse conditions, and improve the tolerance of *L. perenne* to pollution.

## 5. Conclusions

This study indicated that AMF inoculation significantly enhanced the growth and stress resistance of *L. perenne* in Cd-contaminated soils. AMF inoculation improved plant biomass, promoted antioxidant enzyme activities, and regulated the composition of the rhizosphere bacterial community. Additionally, AMF treatment increased nutrient availability in the rhizosphere soil and reduced Cd toxicity in the aboveground parts of the plant, thereby promoting Cd accumulation in the roots. Moreover, AMF facilitated the enrichment of beneficial microorganisms, further enhancing plant resistance to Cd stress. These results suggest that AMF inoculation is an effective method for improving plant adaptability to Cd pollution. Further studies are needed to identify rhizobacterial species that are more responsive to host plants for plant stability. Systematic studies are also required to understand host–AMF compatibility and the mechanisms by which AMF contributes to enhanced plant stability.

## Data Availability

The raw data supporting the conclusions of this article will be made available by the authors on request.

## Appendix A

**Table A1:** Overall display of the rhizospheric bacterial community of *L. perenne*.

**Phylum**	**Class**	**Order**	**Family**	**Genus**	**Species**	**OTU**
36	106	237	388	680	1313	3182
